# Reproductive and Lifestyle Characteristics of Kenyan Women Presenting With Precancerous Cervical Lesions: A Hospital-Based Case–Control Study

**DOI:** 10.24248/EAHRJ-D-19-00002

**Published:** 2019-11-29

**Authors:** Esther Muitta, Tom Were, Anthony N Kebira

**Affiliations:** a Department of Medical Laboratory Sciences, School of Medicine, Mount Kenya University, Thika, Kenya; b Department of Medical Laboratory Sciences, School of Public Health, Biomedical Science and Technology, Masinde Muliro University of Science and Technology, Kakamega, Kenya; c Department of Microbiology, School of Pure and Applied Sciences, Kenyatta University, Nairobi, Kenya

## Abstract

**Background:**

Cervical cancer is a leading cause of cancer in women, accounting for 68% of cancer-related deaths among women in developing countries. Several reproductive, lifestyle and demographic risk factors are associated with increased risk for cervical cancer. This study examined the association of risk factors with precancerous cervical lesion grade in women attending Nakuru County Referral Hospital.

**Methods:**

This hospital-based, case-control study was conducted among women aged 20 to 70 years from January to December, 2017. A total of 142 women were recruited into the study and stratified based on precancerous cervical lesion grades based on the Bethesda System as: atypical glandular cells or adenocarcinoma in situ (AGC/AIS, n=8), high-grade squamous intraepithelial lesions (HSIL, n=59), low-grade squamous intraepithelial lesions (LSIL, n=35), and controls (n=40). Structured questionnaires were used to collect information on demographic, reproductive health, and lifestyle characteristics; anthropo-metric assessments were conducted. Endocervical swabs and scrapings were obtained from the study participants and used for human papillomavirus (HPV)-16/18, and Pap smear screening.

**Results:**

Age differed significantly among the study groups, with age rising with higher grade of precancerous lesion. Higher rates of HPV 16/18 infection was associated with presenting with AGC/AIS (n=8, 100.0%), HSIL (n=47, 79.7%), and (n= 29, 82.9%), compared to controls (n=4, 10.0%; *P*<.001). History of concomitant lower abdominal pain, vaginal bleeding and discharge was associated with higher risk of precancerous lesion in the HSIL group (odds ratio [OR] 8.9; 95% confidence interval [CI], 2.6 to 30.6) and the LSIL group (OR 5.8; 95% CI, 1.8 to 18.8). Bust circumference <99 cm was associated with higher risk of having AGC/AIS (OR 17.4; 95% CI, 1.1 to 276.0), HSIL (OR 5.9; 95% CI, 2.0 to 17.1), and LSIL (OR 2.7; 95% CI, 0.9 to 7.8). Waist circumference <86 cm was associated with higher risk of HSIL (OR, 5.4; 95% CI, 1.9 to 15.4) and LSIL (OR 2.9; 95% CI, 0.9 to 8.2). Having a healthy diet was associated with higher odds of LSIL (OR, 4.2; 95% CI, 1.4 to 12.9), but was not associated with HSIL or AGC/AIS.

**Conclusion:**

This study suggests that high-risk HPV 16/18 infection, chronic lower abdominal pain with vaginal bleeding, and decreased upper and lower trunk body mass, are associated with higher risk of precancerous cervical lesions. Integrating targeted cervical cancer screening in routine reproductive health-care services may reduce the risk of developing cervical cancer.

## INTRODUCTION

Globally, cervical cancer is the fourth most common cancer in women, with an estimated 570,000 new cases and 311,000 deaths in 2018. The burden of cervical cancer is particularly high in sub-Saharan Africa, where it ranks among the top 2 most commonly diagnosed cancers in women, and is a leading cause of cancer-related deaths.^1–3^ In Kenya, over 2000 cervical cancer cases are diagnosed per 100,000 women annually, resulting in an estimated 8,600 deaths in the country each year.^4–10^

Cervical intraepithelial lesions are the precancerous condition of the cervix, which if left untreated, can develop to cervical cancer. These lesions are characterised by abnormal cellular morphology and are detectable by microscopic examination of cervical epithelial cells obtained through Pap smears. The Bethesda System (TBS) is used to grade the severity of abnormal cell morphology. The most common manifestation of precancerous cervical lesions are low-grade squamous intraepithelial lesion (LSIL) and high-grade squamous intraepithelial lesion (HSIL) both of which both are treatable by cervical ablation, cryotherapy or loop electro-excision procedure (LEEP).^11–16^ If left untreated, lesions may progress, and infiltrate adjacent tissue, at which point the disease is diagnosed as an invasive squamous cell carcinoma or, more rarely, a glandular cell cervical cancer (adenocarcinoma).^12^ At this stage, management of lesions is more difficult, and a total abdominal hysterectomy may be recommended in tandem with radiotherapy and/or chemotherapy treatments if metastasis has occurred.^17^

A number of reproductive, lifestyle and demographic risk factors are associated with increased risk for development of cervical cancer. HIV-induced immunodeficiency predisposes women to develop precancerous cervical lesions by enhancing gene expression of oncogenic human papillomavirus (HPV) strains such as high-risk (HR) HPV I6/18,^5^ with immunosuppression increasing risk of progression to cancer.^6–9^ Like HIV, HPV is sexually transmitted, and risk of cervical cancer is associated with sexual risk factors including multiplicity of sexual partners, early sexual debut, high parity and long-term use of hormonal birth control.^5–10^ Demographic factors that have been associated with increased risk of cervical cancer include alcohol and tobacco use, low education, and low socioeconomic status.^7,9^ Malnutrition and wasting may also contribute to increased risk of precancerous cervical lesions in individuals with HPV.^13,14^

To address the high burden of cervical cancer in the country, in June 2019, the Kenyan government implemented mass HPV vaccination, as recommended by many studies, including a 2019 study conducted in Nakuru County, Kenya.^10^ However, many Kenyan women are already infected with HPV, and remain at risk for development of cervical cancer. In the absence of routine screening for cervical intraepithelial lesions though Pap smear, these women may have precancerous cervical lesions that could progress over time.^18,19^ To identify subsets of women at greatest risk of cervical cancer, this study examined the association of reproductive health and lifestyle practices with precancerous cervical lesion grades in women attending Nakuru County Referral Hospital (NCRH).

## METHODS

### Study Site

The study was conducted at the Reproductive Health Services Department of the Maternal and Child Health (MCH) Unit at NRCH, a 250-bed capacity referral hospital in Nakuru County, Kenya serving a population of about 1.2 million residing in the city of Nakuru, its suburbs and rural environs.^20^ The MCH Unit provides medical services for maternal health, including reproductive health, birth control and pre and post natal services. Monthly outpatient attendance at the MCH unit ranges from 150 to 200 women.

### Study Design and Selection of Study Participants

This hospital-based, case-control study assessed the relationship of reproductive and lifestyle factors with precancerous cervical lesion grades in women attending outpatient reproductive health services at the MCH Unit of NRCH from January to December of 2017. Four groups of women were recruited into the study, based on findings from cervical visual inspection with acetic acid and lugol's iodine (VIA/VILI test). The groups were: (1) women with atypical glandular cells or adenocarcinoma in situ (AGC/AIS, n=8), (2) women with high squamous intraepithelial lesions (HSIL, n=59), (3) women with low squamous intraepithelial lesions (LSIL, n=35), and (4) control women in whom no abnormal cellular morphology was observed. Health-seeking women were recruited from the MCH on clinic visit days where suitability to participate was first ascertained through an informed consent process. Enrolment of study subjects was purposively done through the enforcement of predetermined inclusion criteria, which successfully admitted 208 study participants. Data from 142 out of 208 study participants were considered. Sixty-six enrollees' data were invalidated and disregarded during data cleaning, for a variety of reasons, which included invalid questionnaire response entries and entries made from participants with unsuitable human specimens for laboratory analysis. All women seeking reproductive health services from the MCH on clinic visit days where screened for eligibility and exclusion criteria. All study participants were above the age of 20 years and had pretested positive using cervical visual inspection with acetic acid and Lugol's iodine (VIA/VILI). Pregnant women, in addition to those who were unwilling to participate or did not meet the inclusion criteria, were excluded. VIA/VILI-positive women were purposively targeted, as it was believed that their diagnosis at this stage was an indication that sexual debut had occurred.

### Sampling Procedure and Sample Size

An operationalised sample size, calculated from data obtained on cervical cancer burden statistics—which considered figures from Nakuru county as well from the Kenyan cancer registry pilot preliminary records—was estimated using the formulat described by Kothari et al^1,2,20,21^:

nf=n/(1+n/N)

We applied this formula assuming pairwise comparisons between controls and each category of precancerous lesions, and that the ratio between controls and cases would be approximately 1. Our minimum OR of interest was 3.5, and we assumed that explanatory factors would be present in 25% to 75% of the control group. Using this formula we obtained a minimum sample size of about 40 per group. The study included a total of 142 participants, including 40 controls, 35 women in the LSIL group, 59 women in the HSIL group and 8 women in the AGC/AIS group.

### Data Collection and Laboratory Investigations

Data collection procedures were conducted by clinical staff, including nurses, pathologists and cytotechnicians from Nakuru County Referral Hospital; all study staff underwent a structured protocol training prior to study commencement. Information on participant demographics, reproductive health and lifestyle practices, diet and physical exercise were captured in 1-on-1 interviews conducted in the national language of English and translated to Swahili when necessary, with study participants using a structured questionnaire. If a participant did not understand a question, or provided an ambiguous response, the interviewer probed for greater depth or clarity using structured questionnaire probes, which were designed based on validated probes from similarly designed studies.^9,22,23^ Information on diet was obtained through questions regarding weekly food intake using a checklist of food items common in Nakuru County. Study participants were also asked questions regarding the type, frequency and intensity of their physical activity on weekly basis. Questions and categorisations regarding diet and physical activity were developed based on published methodologies of studies collecting similar data.^24,25^ Study participants underwent anthropometric assessment including measurement of body weight, height, mean-upper-arm circumference (MUAC), bust girth and waist circumference. Data on body weight in kilograms and height in meters were used to calculate the body mass index (BMI).

Both cervical swabs and scrape specimens were obtained. Swabs were collected for STI screening (HR HPV 16/18, for example), while scrapes were collected for cellular material in Pap smear preparations that were stained and examined for atypical diversity. Briefly, scrapes were excavated from the cervical wall using a cervical brush; scraped tissue embedded in the brush was smeared onto microscopic glass slides, fixed while wet, stained after drying using Papanicalou staining techniques and dyes (Heamatoxylin, EA 50 and OG 6) and mounted.^26^ Smears were microscopically examined for the presence of abnormal cellular morphology and graded using TBS.^18,27^ Study participants were categorised into 4 discrete cytology categories based on their TBS score as: control (no cytological abnormality), LSIL, HSIL and AGC/AIS. All smears positive for cervical cancer (AGC/AIS) were confirmed by an independent clinical cytologist. All cervical specimens underwent assessment for HR HPV 16/18 using a commercial kit (StrongStep^®^ HPV 16/18 Antigen Rapid Test Device, Limingo Bio), that detects viral antigens.^28^

### Data Analysis

Study questionnaires were data entered into into Microsoft Office Excel (Microsoft Corp., Redmond, WA, USA) and then exported into IBM SPSS Statistics for Windows version 21.0 (IBM Corp, Armonk, NY, USA), where data were checked for presence of outliers and data errors. Bivariate analyses were conducted to assess associations between reproductive health and lifestyle factors and cervical cytology category. In bivariate analyses, Kruskal–Wallis and analysis of variance (ANOVA) tests were used to test for differences in continuous variables across cytology category; chi-square and Fisher's exact tests were used to test for differences across categorical variables. Multivariate logistic regression modeling analyses were performed to identify reproductive health indicators of precervical cancer grade outcome for HR HPV 16/18, clinical history of lower abdominal pain and vaginal bleeding, parity, and multiplicity of sexual partners. Precervical cancer grade outcomes of LSIL, HSIL, and AGC/AIS were entered as the dependent variables; predictors were HR HPV 16/18-positive outcomes, the manifestations of a clinical history of lower abdominal pain and vaginal bleeding, having more than 2 children, and having more than 1 sexual partner with references of the nonclinical manifestations of pain and bleeding history, having less than 2 children, and having less than 1 sexual partner, respectively. The confounding effects of age, birth control method, cervical cancer disease awareness, marital status, and education level were controlled for in the regression model. Data are presented as ORs and 95% CIs. The β coefficient indicates the degree of association differences for the model.

All independent variables that associated significantly by use of the Kruskal–Wallis test (*P* ≤.01) with the dependent variable (precervical cancer status indicated by LSIL, HSIL, or AGC/AIS precancer grades) were further additionally analysed using Dunn's post hoc correction for between-group comparisons (multiple comparisons) to assess which specific precancer group significantly differed by ANOVA (Kruskal–Wallis test) (Table 5).

### Ethical Considerations

Written informed consent was conducted in all study participants prior to carrying out any study procedures. Regardless of their willingness to consent, women with abnormal cytology results were counseled and advised to seek further clinical management from the health-care providers in the MHC clinic. Referral to specialists for symptomatic participants was advised for further management. Ethical approvals were obtained from Kenyatta University Ethical Research Committee (KUERC-KU/R/COMM/51/228; PKU/141/I124) and the Kenya National Council of Science Technology and Innovation (NACOSTI-NACOSTI/RCD/12A/013/148). Institutional approval was provided by the Nakuru County Referral Hospital (RII/VOL.I/08). Special coding for participant data was applied to maintain participant anonymity.

## RESULTS

In total, 142 women were enrolled in the study. Most women were married, had not completed secondary education, and were employed in the informal sector (Table 1). Several demographic variables were associated with detection of abnormal cytology in bivariate analysis. Women presenting with AGC/AIS had a significantly higher median age in years (65; range, 50 to 70 years) years compared to women with HSIL (42; range, 27 to 63 years)), LSIL (38; range, 20 to 57 years years), and controls (34; range, 21 to 55 years; *P*<.001) (Table 1). The proportion of the women with less than a secondary education was higher in the AGC/AIS (n=5, 62.5%) and HSIL groups (n=43, 72.9%) compared to the LSIL (n=17, 48.6%) and control groups (n=21, 52.5%; *P*=.079). The distribution of occupational types and marital status were similar across cervical cytology groups.

**TABLE 1.** Characteristics of the Study Participants (N=142)

Characteristic	Controls, n=40 n (%)	LSIL, n=35 n (%)	HSIL, n=59 n (%)	AGC/AIS^a^, n=8 χ^2^, n (%)	Degrees of Freedom	*P* Value^b^
Median age, years (range)	34 (21–55)	38 (20–57)	42 (27–63)	65 (50–70)		<.001
Education						
≤Primary	21 (52.5)	17 (48.6)	43 (72.9)	5 (62.5)	10.226, 3	.017
>Secondary	19 (47.5)	18 (51.4)	16 (27.1)	3 (37.5)		
**Occupation**^c^						
Informal sector	21 (52.5)	15 (42.9)	33 (55.9)	6 (75)	2.408, 6	.879
Small businesses	15 (37.5)	15 (42.9)	19 (32.2)	2 (25)		
Formal employment	4 (10)	5 (14.3)	7 (11.9)	0 (0)		
**Marital status**						
Married	30 (75)	21 (60)	35 (59.3)	3 (37.5)	2.940, 3	.401
Single	10 (25)	14 (40)	24 (40.7)	5 (62.5)		
**HR HPV16/18**						
Yes	4 (10)	29 (82.9)	47 (79.7)	8 (100)	62.681, 3	<.001
No	36 (90)	6 (17.1)	12 (20.3)	0 (0)		
**History of simultaneous lower abdominal pain, vaginal bleeding, and vaginal discharge**						
Yes	30 (75)	33 (94.3)	56 (94.9)	8 (100)	11.926, 3	.008
No	10 (25)	2 (5.7)	3 (5.1)	0 (0)		
**History of lower abdominal pain and vaginal bleeding only**						
Yes	20 (50)	30 (85.7)	54 (91.5)	8 (100)	27.553, 3	<.001
No	20 (50)	5 (14.3)	5 (8.5)	0 (0)		
**History of lower abdominal pain and vaginal discharge only**						
Yes	25 (62.5)	25 (71.4)	41 (69.5)	6 (75)	0.938, 3	.816
No	15 (37.5)	10 (28.6)	18 (30.5)	2 (25)		
**Birth control use**						
Hormonal	18 (45)	18 (51.4)	33 (56)	0 (0)	21.993, 6	.001
Non-hormonal	16 (40)	10 (28.6)	14 (23.7)	0 (0)		
None	6 (15)	7 (20)	12 (20.3)	8 (100)		
**Parity**						
≥2	22 (55)	19 (54.3)	37 (62.7)	7 (87.5)	1.834, 3	.001
<2	18 (45)	16 (45.7)	22 (37.3)	1 (12.5)		
**Number of sexual partners**						
>1	10 (25)	13 (37.1)	30 (50.8)	2 (25)	6.776, 3	.079
≤1	30 (75)	22 (62.9)	29 (49.2)	6 (75)		
**Knowledge of cervical cancer cause**						
Don't know	20 (50)	24 (68.6)	32 (54.0)	2 (25)	25.843, 9	.002
Infections	7 (17.5)	4 (11.4)	7 (11.9)	0 (0)		
Poor hygiene	4 (10)	7 (20)	18 (30.5)	6 (75)		
Witchcraft	9 (22.5)	0 (0)	2 (3.4)	0 (0)		

Data are presented as frequency, n (percentage, %), unless otherwise indicated;

a Merged group including participants presenting with both precervical cancer grade AGC and AGC/AIS, since only 1 participant was found to have precancer of AIS.

b Differences in age across the study groups were compared using the Kruskal–Wallis test. Comparisons in categorical variables were performed using the Pearson's chi-square test for proportions or Fisher's exact test;

c All participants who identified themselves as housewives (unemployed) concurrently responded that they were involved in small-scale enterprise.

Abbreviations: AGC, atypical glandular cells; AIS, adenocarcinoma in situ; HSIL, high-grade squamous intraepithelial lesion; LSIL, low-grade squamous intraepithelial lesion

In bivariate analysis, several reproductive risk factors were more frequent in women with abnormal cervical cytology compared to controls, with frequency increasing with higher category of abnormality. History of simultaneous lower abdominal pain, vaginal bleeding, and discharge was significantly more prevalent in the AGC/AIS (n=8, 100.0%), HSIL (n=56, 94.9%), and LSIL (n=33, 94.3%) compared with controls (n=30, 75.0%; *P*=.008). Similarly, history of lower abdominal pain with vaginal bleeding was significantly more prevalent in the AGC/AIS (n=8, 100.0%), HSIL (n=54, 91.5%), and LSIL groups (n=30, 85.7%) compared to controls (n=20, 50.0%; *P*<.001). All women in the AGC/AIS group (n=8, 100.0%) reported not using any birth control methods, compared to a minority of women in the HSIL group (n=12, 20.3%), the LSIL group (n=7, 20.0%) and the control control (n=6, 15.0%; *P*=.001). Parity ≥2 was higher among women in the AGC/AIS (n=7, 87.5%) and HSIL (n=37, 62.7%) groups compared to the LSIL (n=19, 54.3%) and control groups (n=22, 55.0%; *P*=.001). Frequency of multiple sexual partners varied across cervical cytology group, though differences did not achieve statistical significance. Having multiple sexual partners was most common among women in the HSIL (n=30, 50.8%) and LSIL groups (n=13, 37.1%), compared to women in the AGC/AIS (n=2, 25%) and control groups (n=10, 25.0%; *P*=.079). Prevalence of HR HPV 16/18 was over 80% among women with abnormal cervical cytology, with the highest prevalence in the AGC/AIS group (n=8, 100.0%) compared to the HSIL group (n=47, 79.7%), the LSIL group (n=29, 82.9%) and the control group (n=4, 10.0%; *P*<.001).

In bivariate analysis of anthropometric measurements, diet and activity revealed a number of differences across the cervical cytology groups. Median weight in kilograms was higher in the AGC/AIS group (73.0; range, 52.0 to 86.0 kilograms), compared to HSIL (70.0; range, 44.0 to 96.0 kilograms), LSIL (70.0; range, 45.0 to 70.0 kilograms) and controls groups (71.0; range, 45.0 to 89.0 kilograms; *P*=.036) (Table 3). Median bust girth in centimeters (cm) was lower in the AGC/AIS (median, 96.0; range, 76.0 to 101.0 cm), HSIL (98.0; range, 74.0 to 126.0 cm), and LSIL (100.0; range, 70.0 to 122.0 cm) groups compared to the control group (73.0; range, 52.0 to 86.0 cm; *P*=.004). Likewise, median waist circumference in centimetres was lower in women with AGC/AIS (74.0; range, 64.0 to 90.0 cm), HSIL (83.0; range, 56.0 to 112.0 cm), and LSIL (86.0; range, 54.0 to 114.0 cm) compared to controls (90.0; range, 51.0 to 109.0 cm; *P*=.002). Height, BMI and MUAC were similar across the cervical cytology groups. In all cervical cytology groups, 80% to 90% of women reported engaging in physical activity. The proportion of women with a healthy diet was was higher in the AGC/AIS (n=5, 62.5%) and HSIL (n=39, 66.0%) groups compared to the LSIL (n=11, 31.4%) and control (n=13, 33.0%; *P*=.004) groups. The proportion of women reporting use of alcohol or tobacco use was low and did not vary significantly across the cervical cytology groups.

In logistic modelling, detection of HR HPV 16/18 was associated with a significantly higher risk of AGC/AIS (odds ratio [OR] 1.947; 95% confidence interval [CI], 1.940 to 1.947), HSIL (OR 36.3; 95% CI, 9.5 to 139.5), or LSIL (OR 50.1; 95% CI, 11.9 to 208.9) (Table 2). Likewise, a history of concomitant lower abdominal pain and vaginal bleeding was associated with higher risk of presenting with AGC/AIS (OR 1.0; 95% CI, 1.0 to 1.0; *P*<.001), HSIL (OR 8.8; 95% CI, 2.6 to 30.6), and LSIL (OR 5.8; 95% CI, 1.8 to 18.7). Regression analyses also illustrated that high odds of having AGC/AIS (OR 17.4; 95% CI, 1.1 to 276.0), HSIL (OR 5.9; 95% CI, 2.0 to 17.1), and LSIL (OR 2.7; 95% CI, 0.9 to 7.8) were associated with a bust circumference <99 cm compared to a bust circumference >99 cm (Table 4). In addition, waist circumference <86 cm was associated with a higher odds of HSIL (OR, 5.4; 95% CI, 1.9 to 15.4) and LSIL (OR 2.9; 95% CI, 0.9 to 8.2). Having a healthy diet was associated with higher odds of LSIL (OR, 4.2; 95% CI, 1.4 to 12.9), but was not associated with HSIL or AGC/AIS.

**TABLE 2.** Multivariate Logistic Regression Analysis of Reproductive Risk Factors and Cervical Lesion Groups

Characteristic	β	OR	95% CI	*P* Value
**HR HPV 16/18**				
LSIL	3.913	50.055	11.993–208.913	<.001
HSIL	3.592	36.323	9.456–139.525	<.001
AGC/AIS	21.390	1.947	1.940–1.947	<.001
**History of simultaneous lower abdominal pain, vaginal bleeding and discharge**				
LSIL	1.758	5.800	1.795–18.739	.003
HSIL	2.183	8.873	2.576–30.561	.001
AGC/AIS	25.347	1.019	1.000–1.019	<.001
**Parity ≥2**				
LSIL	−0.216	0.806	0.270–2.408	.699
HSIL	−0.654	0.520	0.178–1.515	.231
AGC/AIS	0.465	1.592	0.034–74.450	.813
**Multiple sex partners >1**				
LSIL	0.125	1.134	0.256–5.016	.755
HSIL	1.350	3.859	0.905–16.450	.068
AGC/AIS	−0.602	0.548	0.013–23.896	.216

Multivariate logistic regression model analyses were performed to identify reproductive health indicators of precervical cancer grade outcome for HR HPV 16/18, clinical history of lower abdominal pain and vaginal bleeding, parity, and multiplicity of sexual partners. Precervical cancer grade outcomes of LSIL, HSIL, and AGC/AIS were entered as the dependent variables; predictors were HR HPV 16/18-positive outcomes, the manifestations of a clinical history of lower abdominal pain and vaginal bleeding, having more than 2 children, and having more than 1 sexual partner with references of the nonclinical manifestations of pain and bleeding history, having less than 2 children, and having less than 1 sexual partner, respectively. The confounding effects of age, birth control method, cervical cancer disease awareness, marital status, and education level were controlled for in the regression model. Data are presented as ORs and 95% CIs. The β coefficient indicates the degree of association differences for the model. Abbreviations: AGC, atypical glandular cells; AIS, adenocarcinoma in situ; CI, confidence interval; HR HPV, high-risk human papillomavirus; HSIL, high-grade squamous intraepithelial lesion; LSIL, low-grade squamous intraepithelial lesion; OR, odds ratio

**TABLE 3.** Anthropometric, Diet and Physical Activity Characteristics of the Study Participants

Characteristic	Controls, n=40 n (%)	LSIL, n=35 n (%)	HSIL, n=59 n (%)	AGC/AIS, n=8 n (%)	χ^2^, Degrees of Freedom	*P* value -
**Weight^b^, kg**	71 (45–89)	70 (45–70)	70 (44–96)	73 (52–86)	–	.0348
**Height^b^, m**	1.6 (1.5–1.8)	1.6 (1.5–1.8)	1.6 (1.5–1.8)	1.6 (1.5–1.6)	–	.763
**BMI^b^, kg/m^2^**	26 (19–34.0)	25 (20–35)	24 (16–33)	27.5 (21–33)	–	.570
**MUAC^b^, cm**	33 (23–45)	31 (20–44)	30 (21–42)	32 (23–34)	–	.110
**Bust^b^, cm**	103 (78–122)	100 (70–122)	98 (74–126)	96 (76–101)	–	.0035
**Waist^b^, cm**	90 (51–109)	86 (54–114)	83 (56–112)	74 (64–90)	–	.0045
**Physical activity**						
Yes	22 (80.0)	33 (94.3)	56 (94.9)	8 (100)	7.679, 3	.050
No	8 (20.0)	2 (5.7)	3 (5.1)	0 (0)		
**Diet**						
Healthy	13 (33.0)	11 (31.4)	39 (66)	5 (62.5)	13.175, 3	.004
Unhealthy	27 (70.0)	24 (68.6)	20 (34)	3 (37.5)		
**Ever used alcohol**						
Yes	3 (7.5)	3 (8.6)	6 (10.2)	1 (12.5)	0.902, 3	.825
No	37 (92.5)	32 (91.4)	53 (89.8)	7 (87.5)		
**Ever used tobacco**						
Yes	1 (2.5)	2 (5.7)	6 (10.2)	1 (12.5)	3.440, 3	.328
No	39 (97.5)	33 (94.3)	53 (89.8)	7 (87.5)		

Data presented are presented as frequency, n (percentage, %), of subjects, unless otherwise indicated. Across-group comparisons for continuous variables were performed using the Kruskal–Wallis test. Comparisons in categorical variables amongst the study groups were performed using the chi-square test for proportions.

a Merged group including participants presenting with both precervical cancer grade AGC and AGC/AIS, since only 1 participant was found to have precancer of AIS.

b Data presented as median (range)

**TABLE 4.** Multivariate Logistic Regression Analysis of Anthropometric Characteristics and Diet Practices

Characteristic	β	Odds Ratio^a^	95% CI	*P* Value
**Body weight (median ≤68 kg)**				
LSIL	−0.087	0.9	0.3–2.5	.863
HSIL	0.808	2.2	0.8–5.9	.103
AGC/AIS	−1.839	0.2	0.0–5.8	.317
**Bust girth (median ≤99 cm)**				
LSIL	0.974	2.7	0.9–7.8	.077
HSIL	1.767	5.9	2.0–17.1	.001
AGC/AIS	2.858	17.4	1.1–276.1	.043
**Waist circumference (median ≤86 cm)**				
LSIL	1.052	2.9	0.9–8.2	.051
HSIL	1.681	5.4	1.9–15.4	.002
AGC/AIS	1.816	6.2	0.5–80.2	.166
**Unhealthy diet**				
LSIL	−1.433	4.2	1.363–12.881	.012
HSIL	−0.90	1.1	0.391–3.062	.864
AGC/AIS	3.108	0.0	0.000–4.065	.177

Multivariate logistic regression analyses were performed to identify anthropometric and diet indicators of precervical cancer grade outcome for body weight, bust girth, waist circumference, and unhealthy diet consumption. Precervical cancer grade outcomes of LSIL, HSIL, and AGC/AIS were entered as the dependent variables, and predictors of weight medians ≤68 kg, bust medians ≤99 cm and waist medians ≤86 cm, including median values above the stated, as references in the regression model. Unhealthy diet was also entered as a predictor with healthy diet serving as the reference. The confounding effects of age, birth control method choice, cervical cancer disease awareness, marital status, and education level were controlled for in the regression model. Data are presented as OR and 95% CI. The β coefficient indicates the degree of association differences for model. Abbreviations: AGC, atypical glandular cells; AIS, adenocarcinoma in situ; CI, confidence interval; HR HPV, high-risk human papillomavirus; HSIL, high-grade squamous intraepithelial lesion; LSIL, low-grade squamous intraepithelial lesion; OR, odds ratio

Dunn's post hoc corrections were performed on significantly different variables from the Kruskall–Wallis ANOVA (age, weight, bust measurement, and waist circumference) (Table 5).

**TABLE 5.** Dunn's Post Hoc Analysis for Between-Group Analysis of Variance Differences

Characteristic	Age (median >38 years)	Weight (median ≤68 kg)	Bust (median ≤99 cm)	Waist (median ≤86 cm)
**Kruskal–Wallis**	*P*<.001	*P*=.0348	*P*=.0035	*P*=.0045
**In between group median variance**	*P*<.05	*P*<.05	*P*<.05	*P*<.05
**AGC/AIS vs HSIL**	*P*<.01	–	–	–
**AGC/AIS vs LSIL**	*P*<.001	–	–	–
**AGC/AIS vs Control**	*P*<.001	–	–	–
**HSIL vs LSIL**	–	–	–	–
**HSIL vs Control**	*P*<.001	–	*P*<.01	*P*<.01
**LSIL vs Control**	–	–	–	–

## DISCUSSION

This case–control study, conducted at a large, public hospital in Kenya, examined the influence of demographic, reproductive, and lifestyle factors on risk of precancerous cervical lesions. Older women with a low level of education had the highest prevalence of HR HPV 16/18 and were more likely to have the highest grade lesions. Consistent with the sexual mode of HPV transmission, sexual and reproductive risk factors, including multiplicity of sexual partners, multiparity, and lack of birth control use were positively associated with abnormal cervical cytology. Strikingly, we detected HR HPV 16/18 in the cervical specimens of over 80% of women with abnormal cervical cytology, highlighting the importance of this viral strain as a cause of precancerous cervical lesions in Kenya.

Our finding that the median age of women was highest in the AGC/AIS group is consistent with other studies conducted in Africa and Europe,^6,21,22,29–31^ and likely reflects the progression of untreated, precancerous cervical lesions over time. While over 90% of HPV infections clear within 2 years^11,16^ even without treatment, some infections, particularly those of oncogenic viral sub-types 16 and 18, may progress to cancer over a period of 10 to 20 years.^16^ Infection with HR HPV 16/18 strains was common among our study participants, which is consistent with the high prevalence of HR HPV-16/18 (>60%) in the general population in Kenya,^9,22,23^ and rates of detection of over 70% among patients at Kenyatta National Hospital diagnosed with high grade cervical lesions or squamous cell carcinomas.^32^ Thus an accumulation of high grade lesions among older women may reflect the natural history of HPV 16/18 infection in a setting in which screening and treatment for precancerous cervical lesions is largely absent.

We found that over 90% of women with cervical lesions had concurrent lower abdominal pain, vaginal bleeding and vaginal discharge. In logistical analysis these signs and symptoms were associated with high risk for LSIL, HSIL and AGC/AIS grades, with risk increasing across higher category of cytological abnormality. These findings mirror those of a study conducted in Uganda, which found increased incidence of lower abdominal pain discomfort and vaginal bleeding among patients undergoing treatment for high grade precancerous cervical lesions.^33–35^ The combination of abnormal vaginal bleeding and pelvic pain are common early indicators of metastasis of cervical cancer.^35,36^ However, these signs and symptoms were also reported 75% of our study controls, reflecting the low specificity of abdominal pain and vaginal bleeding as criteria for suspicion of pre-cancerous cervical lesions. This lack of specificity underlies current recommendations to screen of all women at risk for cervical cancer, regardless of symptoms.

In our patient population, lack of birth control use and multiparity were significantly associated with detection of precancerous cervical lesions, which is consistent with other studies conducted in Nigeria, the USA and the UK.^8,37–39^ Higher parity may increase the occurrence of ovarian hormone imbalances during and/or following pregnancy.^40^ These imbalances may perturb metaplastic processes during cervical wall development and differentiation in which columnar cells are converted to squamous cells within the transformation zone of parabasal layer of the cervical wall.^19^ Cells undergoing metaplasia are more vulnerable to HPV infection, due to numerous mitotic events which may concurrently propagate HPV DNA transcription in infected cells.^15^

Several studies from Africa, and the U.S. have reported increased risk of cervical cancer in association with having multiple sexual partners.^22,40,41^ However, in logistic analysis controlling for demographic factors, we did not find an association between multiplicity of sexual partners and presence of precancerous cervical lesions. Higher transmission of HR HPV 16/18 strains is thought to underlie the association between multiple sexual partners and increased risk of precancerous cervical lesions or cervical cancer. However, in our study the much lower frequency of HR HPV 16/18 infection among control women was not coupled with a lower prevalence of having multiple sexual partners. We speculate that the participants in this study group were better informed about safe sexual practices denoted by the high proportion of women who had secondary and above level of education relative to participants who manifested HSIL and AGC/AIS lesions. This result mirrors a study in the US which investigated multiplicity of sexual partners and HR HPV infection.^42^

Against our expectation, our analysis revealed that having a healthy diet was more frequent among women in the HSIL and AGC/AIS groups compared to women in the LSIL and control groups. This contrasts with findings from a trial conducted in the U.S. suggesting that high consumption of cruciferous vegetables, which are common in the Kenyan diet, may reduce risk of high grade cervical lesions. In the trial, 12-week oral administration of indole-3-carbinol, a compound found in cruciferous vegetables such as cabbage and kale, was more effective than placebo in regression of precancerous cervical lesions.^43^ Other studies of the association between diet and precancerous cervical lesions are more equivocal. An observational study from India which found no significant difference in 24-hour dietary recall between healthy women and women presenting with LSIL or HSIL.^44^ We speculate that our finding that a healthy diet was more common in women with high grade precancerous lesion may be due to age-related differences in diet, that were not adequately controlled for in analysis.

### Limitations

Our study had several limitations. Due to the retrospective nature of the study, we were unable to evaluate the influence of explanatory factors on progression of precancerous lesions, or report on the frequency of regression. Assessment of diet was based on 1-week recall, and may not have provided a precise estimate of actual food intake. Finally, our sample size was relatively small and may not have allowed for estimation of moderate to small effect sizes.

## CONCLUSION

Protracted clinical histories of lower abdominal pain and heavy vaginal bleeding including recognised reduction of body mass in the upper arms, waist, and bust areas indicating wasting syndrome are important predictors of precervical cancer onset. These signs and symptoms can be applied routinely as valid reasons for conducting a cervical cancer screening when identified in women presenting at the reproductive health clinics, as they may predict precervical cancer development. High median age values suggest that having an advanced age predisposes to precancer predictor signs. Substantial HR HPV 16/18 positivity confirms that this organism is indeed a precursor to precancer development and should be routinely tested or screened for in women seeking reproductive health services.
